# To use or not to use dipyrone? Or maybe, Central Station versus ER? That is the question…

**DOI:** 10.1590/S1516-31802001000600001

**Published:** 2001-11-01

Dipyrone is a kind of pyrazolone derivative sold as an over-the-counter painkiller, which is very popular in Brazil. Its use has become so impregnated in our lives that, in a scene in the movie "Central Station", the character played by the Academy Award nominee Fernanda Montenegro was shown keeping a small bottle of dipyrone in a drawer with her keys and some money. This portrayal was not a chance occurrence. In a recent survey among outpatients of Hospital das Clinicas in São Paulo, Brazil, dipyrone was cited as the number one painkiller by more than 50% of the participants.^1^

Dipyrone is widely used, not only in Brazil but also in other countries such as France, Germany, Hungary, Israel, Spain, Sweden, and Thailand. However, the use of dipyrone was proscribed more than twenty years ago in the United States of America due to its putative role in depressing bone marrow and causing aplastic anemia and agranulocytosis in association with its use. What are the facts (evidence and hearsay) regarding dipyrone?

Dipyrone was developed in Germany many years ago but, as a consequence of its proscription in the USA, few clinical trials have been performed to evaluate either its efficacy or safety as a painkiller in the era of evidence-based medicine. However, ancillary studies addressing the analgesic effect of dipyrone have been made using adequate methodology.

Recently, a paper published by a Brazilian team has shown the positive effect of metamizol (intravenous sodium dipyrone) in acute migraine and tension-type headache treatment in a placebo-controlled clinical trial.^2^ A cooperative trial published in *Cephalalgia* comparing metamizol and aspirin for treatment of episodic tension-type headache has shown that more profound pain relief was provided by 0.5 and 1 g metamizol than did 1 g ASA.^3^

Now we also have various studies from different countries and populations that have evaluated a possible association between dipyrone and blood dyscrasia, using good methodologies. A French study on drug use and aplastic anemia has demonstrated no correlation between this condition and previous use of dipyrone derivatives.^4^ The "International Agranulocytosis and Aplastic Anemia Study" showed up a slightly increased risk for agranulocytosis among dipyrone users in some regions but not in others. However, it is important to point out that the absolute risks detected in that study were very low.^5^ In Thailand, a cooperative study between Thai and American researchers brought out the same result, i.e. no material association between dipyrone derivatives and aplastic anemia was detected.^6^

We now have a Brazilian study on the epidemiology of aplastic anemia and its risk factors. It was a case-controlled study that analyzed several associations between drugs and aplastic anemia, with a particular focus on dipyrone. The results do not show any association between dipyrone use and aplastic anemia.^7^

Although the evidence for possible serious side effects from dipyrone use is weak, a strong lobby against its prescription was launched in the Brazilian lay press.

Considering the pressure, the Brazilian Public Health Surveillance System, Agência Nacional de Vigilância Sanitária (ANVISA), organized a panel with the participation of many Brazilian and international scientists specially invited for the meeting. Based on the evidence available up to the present day, as summarized above, they concluded that the sale of dipyrone as an over-the-counter medication in Brazil could continue. In our opinion, this was the correct decision. It was the first time that Brazilian officials and scientists had worked together to defy an American decision.

There are rumors that there was a potential conflict of interest in the American prohibition of dipyrone, considering that it was a German-developed drug. The rationale is weak, but the emotion is strong. In an episode of the TV series E.R. (Emergency Room) a Mexican-American boy comes to the emergency room with sepsis due to aplastic anemia probably attributable to two drugs prescribed by someone else. Obviously, one of those drugs was dipyrone.

In the light of evidence-based medicine and forgetting E.R. episodes: it is time to take decisions based on evidence and not on prejudices.
